# Kinesin-2 Controls the Motility of RAB5 Endosomes and Their Association with the Spindle in Mitosis

**DOI:** 10.3390/ijms19092575

**Published:** 2018-08-30

**Authors:** Emanuela Pupo, Daniele Avanzato, Marco Scianna, Amanda Oldani, Guido Serini, Letizia Lanzetti

**Affiliations:** 1 Department of Oncology, University of Torino Medical School, 10060 Torino, Italy; daniele.avanzato@ircc.it (D.A.); guido.serini@ircc.it (G.S.); 2 Candiolo Cancer Institute, FPO–IRCCS, Candiolo, 10060 Torino, Italy; 3 Department of Mathematical Sciences, Politecnico di Torino, 10129 Torino, Italy; marco.scianna@polito.it; 4 IFOM, The FIRC Institute for Molecular Oncology Foundation, 20139 Milan, Italy; amanda.oldani@ifom.eu

**Keywords:** RAB5, Kinesin-2, KIF3A, KIF3B, mitosis, nuclear envelope breakdown, vesicular trafficking

## Abstract

RAB5 is a small GTPase that belongs to the wide family of Rab proteins and localizes on early endosomes. In its active GTP-bound form, RAB5 recruits downstream effectors that, in turn, are responsible for distinct aspects of early endosome function, including their movement along microtubules. We previously reported that, at the onset of mitosis, RAB5positive vesicles cluster around the spindle poles and, during metaphase, move along spindle microtubules. RNAi-mediated depletion of the three RAB5 isoforms delays nuclear envelope breakdown at prophase and severely affects chromosome alignment and segregation. Here we show that depletion of the Kinesin-2 motor complex impairs long-range movement of RAB5 endosomes in interphase cells and prevents localization of these vesicles at the spindle during metaphase. Similarly to the effect caused by RAB5 depletion, functional ablation of Kinesin-2 delays nuclear envelope breakdown resulting in prolonged prophase. Altogether these findings suggest that endosomal transport at the onset of mitosis is required to control timing of nuclear envelope breakdown.

## 1. Introduction

The small GTP binding protein RAB5 is required for the biogenesis of early endosomes and identifies this vesicular compartment [1]. Early endosomes are made of vesicles and elongated tubules whose dimensions range from 60 to 400 nm [2]. Several endocytic pathways, bringing different cargoes, plasma membrane receptors, and soluble carriers, converge on this compartment which works as a sorting station where the internalized components can be recycled back to the plasma membrane or sorted to other vesicular compartments (recycling endosomes, late endosomes, Trans Golgi Network) [2,3]. Early endosomes are quite heterogeneous, not only in their morphology, but also in their composition as they display a mosaic of structural domains, made of distinct proteins and lipids, endowed with a number of functions including vesicle budding, fusion, and movement [2,4]. Endosomes are transported along microtubules by kinesin and dynein motor proteins [5]. The dynein–dynactin complex moves endosomes towards the microtubule minus ends, proximal to the microtubule organizing center. Transport to the plus ends relies on kinesin motors [5]. Distinct kinesins have been found so far to move early endosomes: KIF5B [6] and KIF16B [7,8]. In particular, KIF16B moves early endosomes in a process that requires RAB5 and its effector, the phosphatidylinositol-3-OH kinase hVPS34. Overexpression of KIF16B relocates early endosomes to the cell periphery and inhibits transport to the degradative pathway [8].

Here we propose that Kinesin-2 also participates to the motility of this vesicular compartment.

Among the wide family of kinesins, Kinesin-2 is a plus end directed transporter made of two motor subunits, KIF3A and KIF3B, and a cargo-binding subunit, KAP3 [9,10,11]. During embryonic development, Kinesin-2, by translocating multiprotein particles intraciliary, allows the formation of motile primary cilia, which in turn determine the acquisition of left-right asymmetry [11,12,13]. Furthermore, intraflagellar transport by KIF3A is also required for the Hedgehog signal transduction cascade [14]. Beside regulating transport within cilia, Kinesin-2 has been shown to control late endosomes and lysosomes motility since overexpression of a motorless KIF3A mutant affects lysosome positioning and acidification [15]. In cell-free assays that take advantage of purified early and late endosomes and of reconstituted microtubules, this motor complex have also been found to move early endosomes [16]. However, the physiological role of Kinesin-2 in the transport of early endosomes is presently unclear.

We investigated the role of Kinesin-2 in the motility of RAB5 endosomes in living cells and found that it is important for long-range movement of early endosomes in interphase and for the localization of these vesicles within the spindle at mitosis. Functional ablation of Kinesin-2 recapitulates the defects observed upon depletion of RAB5 in nuclear envelope breakdown at the onset of mitosis.

## 2. Results

### 2.1. Kinesin-2 Silencing Affects Long-Range Movement of Early Endosomes in Interphase Cells

During a search for novel interacting partners of USP6NL, a GTPase-activating protein for RAB5 that also works as a RAB5-effector, we identified KIF3A among the putative binding proteins (Supplementary Table S1). The interaction between USP6NL and KIF3A was found using a yeast two-hybrid approach and was not further analyzed. However, this finding prompted us to investigate the involvement of the Kinesin-2 complex, to which KIF3A belongs, in the motility of RAB5 endosomes.

To this end, we generated a U2OS cell population stably expressing YFP-RAB5. Increased RAB5 expression is known to promote activation of this GTPase by elevating the number of molecules that became prenylated, inserted in the membrane, and loaded with GTP [17]. Here, they promote all the phenotypes that are under the control of RAB5, including the homotypic fusion of endosomes [17]. This turns out to be a very useful property for live cell imaging purposes because it increases the size of vesicles that are in part largely below the limit of resolution of most of the live cell imaging techniques (which is approximately 200 nm) [18].

**Figure 1:**
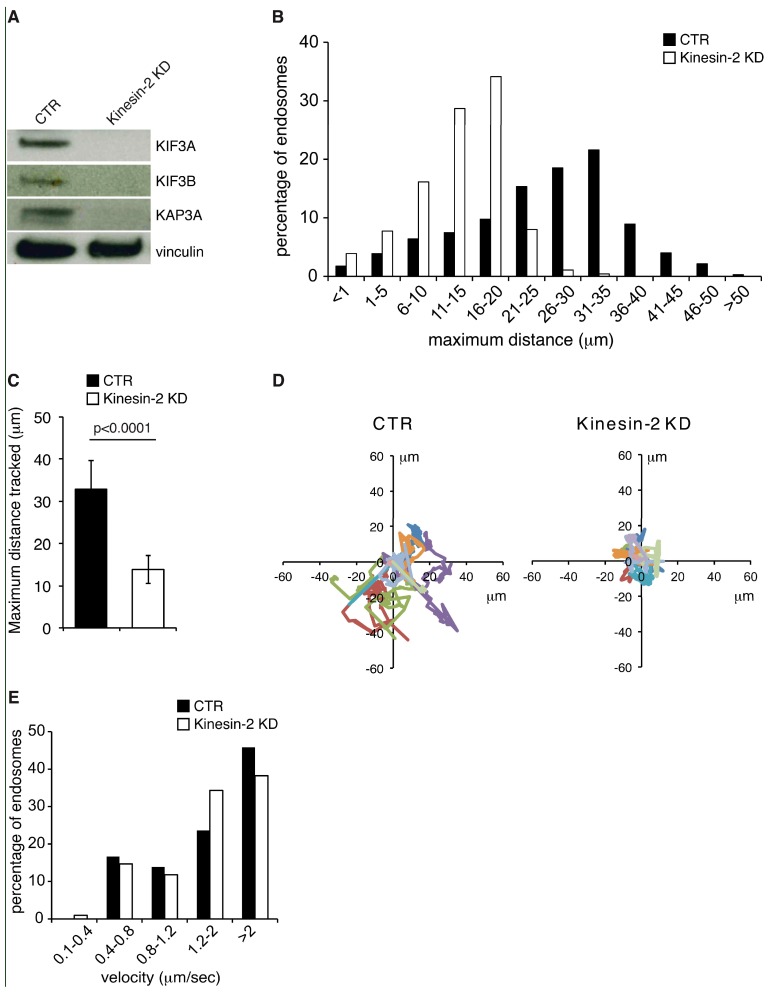
Kinesin-2 silencing affects the motility of RAB5 endosomes. (**A**) Total cellular lysates from U2OS cells silenced with control oligo (CTR) or with KIF3A and KIF3B oligos (Kinesin-2 KD) were immunoblotted as shown on the right. (**B**) Bar graph representing the percentage of endosomes in each of the twelve categories (in μm, indicated below) of maximum distance travelled. Black bars are endosomes from cells silenced with control oligo (CTR), white bars are endosomes from cells silenced with KIF3A and KIF3B oligos (Kinesin-2 KD). Twenty-five endosomes per cell with 10 cells per experiments/condition from three independent experiments were analyzed (total number of endosomes tracked/condition = 750). (**C**) Bar graph representing the mean displacement of YFP-RAB5 endosomes in cell silenced with control oligo (black bar) or with KIF3A and KIF3B oligos (white bar) within two minutes. Means ± SD. Three-hundred endosomes were evaluated in each condition. Ten endosomes in 10 different movies were measured and the experiment was repeated three times. Statistical analysis was done using the *t*-test. (**D**) Tracks of 10 YFP-RAB5 endosomes from a representative cell silenced as indicated on top. (**E**) The velocity of YFP-RAB5 endosomes was evaluated in cells silenced with control oligo (CTR) or with KIF3A and KIF3B oligos (Kinesin-2 KD). At least 72 vesicles per condition from three independent experiments were randomly picked from the movies and analyzed tracking their path using the MetaMorph plugin “Track objects” for 2 min. The bar graph shows the percentage of endosomes in the different velocity categories.

To test the involvement of Kinesin-2 in RAB5 endosome motility we silenced Kinesin-2 in the YFP-RAB5 expressing cells. To achieve complete depletion of the entire Kinesin-2 complex, both the two motor subunit KIF3A and KIF3B were silenced with specific oligos, nontargeting oligo was employed as control (Figure 1A and Material and Methods). Silenced cells were acquired at a speed of 3.83 frames per second for approximately 2 min (Supplementary movies M1 and M2). The high acquisition speed allowed us to track the path travelled by the vesicles and to evaluate their velocity.

Based on the notion that kinesin motors are required for long-range vesicle movements [11,19], we analyzed this parameter in the silenced cells by measuring the maximum displacement of the vesicle from the original position at start within 2 min of videomicroscopy using the Matlab software. Early endosomes display a wide range of different movements. We instructed the software, with computational routines properly encoded in the program, to randomly pick the vesicles and track them excluding from the quantification all the endosomes that merge or split during the time of acquisition. Using these criteria, the program provided the maximum displacement from the original position at start for 750 endosomes in control and Kinesin-2 KD cells (twenty-five endosomes per movie in 10 movies/condition from three independent experiments were measured). Endosomes were then ranked in ten classes according to the maximum displacement reached (progressively increasing from 1 to 50 μm), plus two extra classes that included the endosomes that travelled a distance <1 µm or >50 µm (Figure 1B). In control cells, 56% of the endosomes reached a distance comprised between 21 and 35 μm (Figure 1B). Conversely, in the Kinesin-2 KD cells, the majority of vesicles (approximately 79%) showed a displacement comprised between 6 and 20 μm (Figure 1B). Of note, the percentage of Kinesin-2 depleted vesicles that were able to travel more than 20 µm from the point of origin was severely reduced and almost no endosomes were found to cover distances greater than 35 μm (Figure 1B).

To further characterize the motility of RAB5 endosomes in absence of Kinesin-2, we re-analyzed our movies by excluding all the endosomes that did not move. We considered those endosomes that reach a maximum distance equal or below 5 μm “not moving”, corresponding to the first two classes of Figure 1B. We tracked 300 endosomes both in control and in Kinesin-2 KD cells. The average displacement covered by the endosomes was 32.85 µm in control versus 13.83 µm in Kinesin-2 KD (Figure 1C) indicating that vesicles cannot travel far in absence of Kinesin-2. This can also be appreciated by looking at the paths travelled by 10 representative endosomes from one cell silenced with control or Kinesin-2 oligos tracked by the Matlab program (Figure 1D). Similar tracks are also obtained by the MetaMorph program choosing five vesicles in control and in Kinesin-2 silenced cell from Supplementary movies M1 and M2 (Supplementary Figure S1).

Finally, we analyzed endosomes velocity as described in Gasman et al. [20]. Endosomes were ranked in five classes according to their average velocity. In this case, significant variation in endosomes velocity was not observed upon depletion of Kinesin-2 (Figure 1E) indicating that other motors are responsible for transporting these vesicles. However, this occurs along tracks that do not move too far from the point of origin. Altogether these data indicate that Kinesin-2 participates in the long-range movement of early endosomes.

### 2.2. Depletion of Kinesin-2 Impairs Localization of RAB5 Endosomes to the Mitotic Spindle

We have previously shown that the localization of RAB5 positive endosomes changes during the cell cycle [21]. RAB5 vesicles cluster around spindle poles at late G2/prophase, before nuclear envelope breakdown, and redistribute during prometaphase, populating the spindle region. In metaphase, they move bidirectionally along spindle microtubules [21].

**Figure 2:**
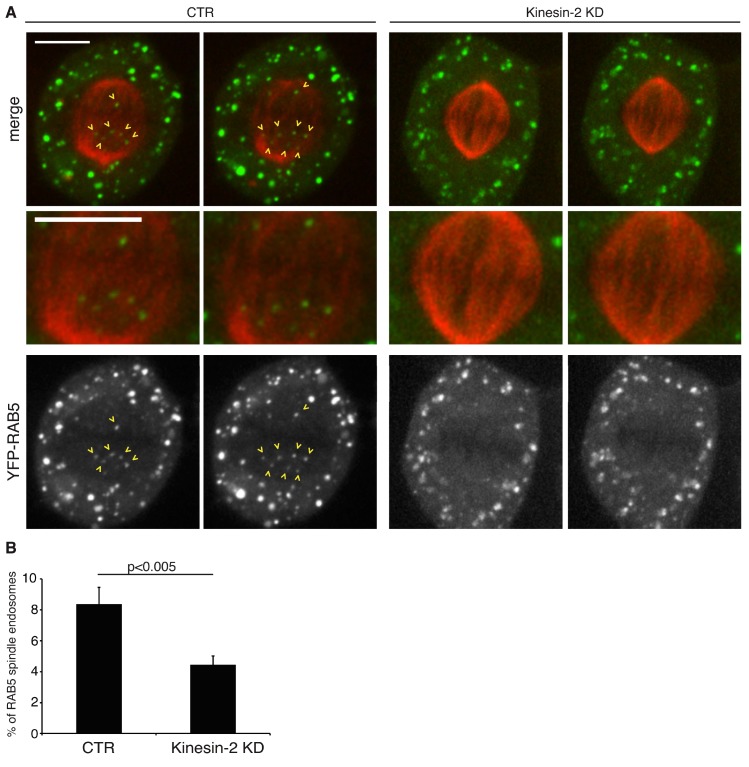
Kinesin-2 silencing reduces the number of RAB5 endosomes that localize to the spindle. (**A**) Two frames have been selected from the movies showing metaphase YFP-RAB5 and RFP-α-tubulin cells, silenced as indicated on top. In the control cell (corresponding to Supplementary movie M3) frame 40 and frame 79 have been selected; in Kinesin-2 KD (Supplementary movie M4) frame 45 and 73 are shown. The images are Z-projections of seven slices (corresponding to 3 μm) taken in the equatorial region of the spindle. The intensity of the red channel has been increased exclusively in this panel in the control cell for visualization purposes. Arrows indicate the RAB5 endosomes located within the spindle in the control cell. No vesicles can be found on the spindle in the Kinesin-2 KD cell. This region of the spindle is also magnified in the panels below. Scale bar here and in the following figures is 10 μm. (**B**) Bar graph representing the percentage of RAB5 positive vesicles at the spindle of cells silenced with control oligo (CTR) or with KIF3A and KIF3B oligos (Kinesin-2 KD). At least 30 movies per condition from three independent experiments were analyzed (mean ± SEM).

Based on the fact that Kinesin-2 takes part in early endosome movement in interphase cells, we investigated whether its depletion might also affect the spindle-associated RAB5 vesicles at metaphase. To this end, U2OS cells stably expressing YFP-RAB5 and RFP-α-tubulin were imaged using time-lapse spinning disk microscopy. Depletion of Kinesin-2 significantly reduced the number of vesicles that localize within the spindle (Figure 2A,B and Supplementary movies M3 and M4). Using an automated macro for quantification, we measured a reduction of approximately 50% in the number of vesicles associated with the spindle in the Kinesin-2 KD cells (Figure 2B). Similar results were obtained on fixed cells stained with anti-α-tubulin antibody (Supplementary Figure S2A,B). To further support this, we noticed that, as previously reported [22], KIF3A diffusely localizes along spindle microtubules showing also a partial overlap with some YFP-RAB5 endosomes (Supplementary Figure S2C).

These findings argue that Kinesin-2 dependent transport is required to localize RAB5 endosomes to the spindle and this occurs before the prometaphase step.

### 2.3. The Mitotic Spindle Is Populated by RAB5 Early Endosomes That Carry the APPL1 Marker

**Figure 3:**
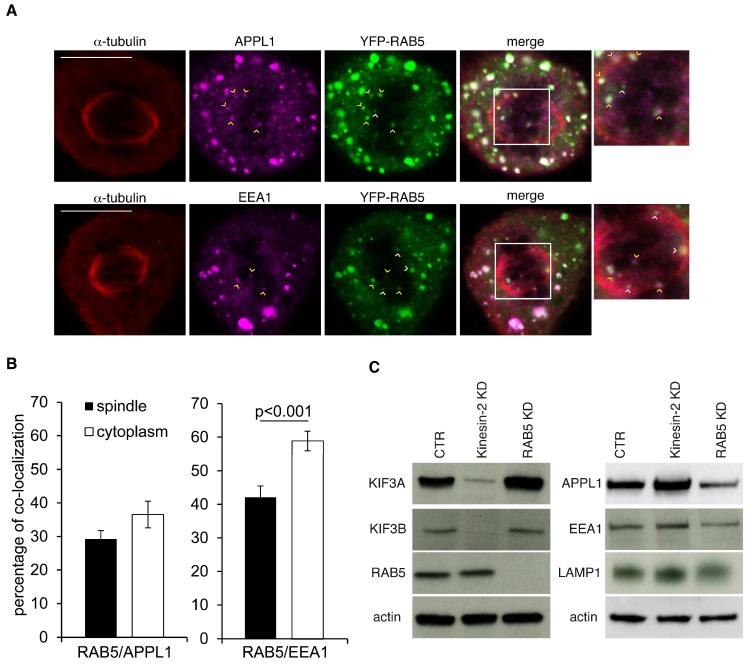
Most of the RAB5 vesicles associated with the spindle are APPL1 endosomes. (**A**) Confocal images of U2OS cells stably expressing YFP-RAB5 (in green) stained with α-tubulin (in red) and APPL1 (in magenta, top row) or EEA1 (in magenta, bottom row). Yellow arrowhead point to RAB5 vesicles associated with the spindle colocalizing with APPL1 or EEA1, white arrowheads to vesicles that do not colocalize. Insets on the right highlight the spindle region. (**B**) Bar graph representing the percentage of colocalization between RAB5 and either APPL1 or EEA1 within the spindle region or the cytoplasm. Twenty-nine cells per condition were analyzed from three independent experiments. Mean values ± SEM. (**C**) Total cellular lysates from U2OS cells silenced with control oligo (CTR), or KIF3A and KIF3B oligos (Kinesin-2 KD), or RAB5 oligos were immunoblotted as indicated on the left.

Early endosomes are characterized by the presence of effectors that bind to the active GTP-bound form of RAB5. Among them, EEA1 and APPL1 have been shown to mark distinct pools of RAB5 endosomes [4]. Thus, to gain insights into the nature of spindle-associated RAB5 vesicles, we analyzed whether they express these two markers measuring the extent of colocalization between YFP-RAB5 and either APPL1 or EEA1. Colocalization between RAB5 and APPL1 was similarly found both on endosomes in the mitotic cytoplasm and in the spindle region (Figure 3A,B). Conversely, overlap between the RAB5 and EEA1 stainings on endosomes that were associated with the spindle was reduced compared to the colocalization observed on the vesicles dispersed in the mitotic cytoplasm. This latter result is also confirmed by previous findings showing that EEA1 endosomes are not moving within spindles [23].

To test whether depletion of Kinesin-2 might affect the composition of RAB5 endosomes, we evaluated the levels of RAB5, its effectors, and of LAMP1, a marker of late endosomes/lysosomes by Western blotting. We found no major alterations on RAB5, EEA1, APPL1, or LAMP1 protein amount in Kinesin-2 KD cells (Figure 3C). Conversely, silencing of the three RAB5 isoforms reduced all these vesicular markers in agreement with findings showing that functional ablation of RAB5 affects the early endosomal compartment as well as the formation of late endosome and lysosomes [1].

### 2.4. Kinesin-2 Silencing Delays Nuclear Envelope Breakdown and Entry into Mitosis

RAB5 controls chromosome congression and progression through mitosis both in mammalian cells and in the Drosophila model organism [21,24]. In human cells, depletion of the three RAB5 isoforms impairs kinetochore maturation and stable attachment by spindle microtubules resulting in delayed metaphase and aberrant chromosome segregation in the daughter cells [21]. These mitotic defects are preceded by a severe delay in nuclear envelope breakdown at prophase [21].

**Figure 4:**
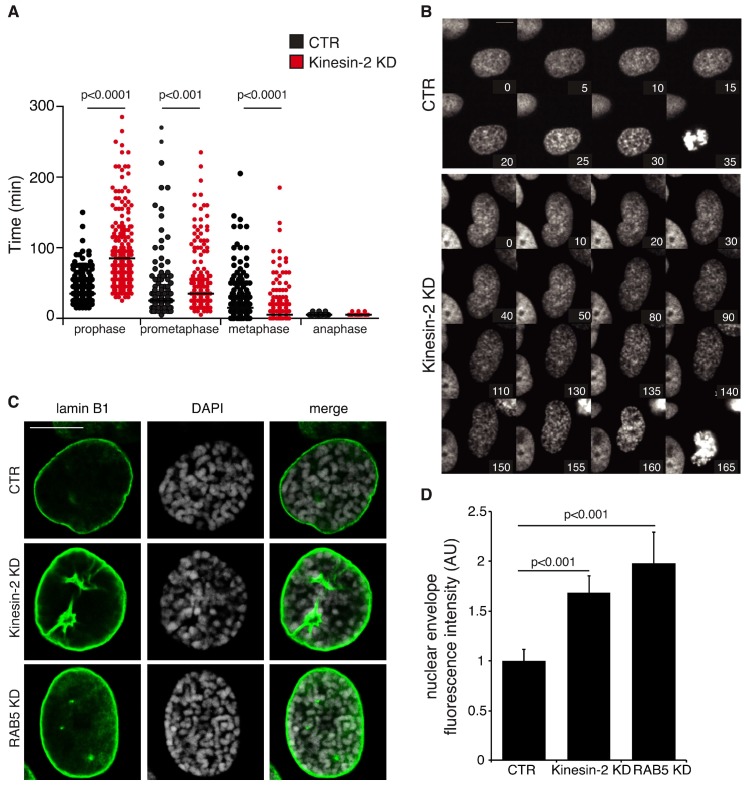
Kinesin-2 depletion delays prophase and nuclear envelope breakdown. (**A**) Scatter plot showing the time spent in the different mitotic phases (indicated below) by U2OS cells silenced with control oligo (CTR, *n* = 198 in black) or with KIF3A and KIF3B oligos (Kinesin-2 KD, *n* = 171, in red) from three independent experiments. (**B**) Selected frames of a single section from movies of cells stably expressing H2B-GFP silenced with control oligos (CTR; Top) or KIF3A and KIF3B oligos (Kinesin-2 KD; Bottom) showing the duration of prophase. Time is in minutes; *t* = 0 is defined as the time point at which chromosome condensation becomes evident. (**C**) Confocal sections of U2OS cells silenced with control oligo (CTR), KIF3A and KIF3B oligos (Kinesin-2 KD), or RAB5 oligos (RAB5 KD) at prophase stained with anti-lamin B1 (green) antibody and DAPI (gray). (**D**) Bar graph of nuclear envelope fluorescent intensity normalized over control in the cells silenced as indicated on bottom. Mean values ± SEM. Thirty-five cells per condition from four independent experiments were analyzed in CTR and Kinesin-2 KD, 19 cells from three independent experiments were analyzed in the RAB5 KD cells.

Based on findings that Kinesin-2 controls the localization of RAB5 endosomes at the spindle, we analyzed whether depletion of Kinesin-2 might reproduce the mitotic defects observed upon impairment of RAB5. To this end, U2OS cells stably expressing H2B-GFP and RFP-α-tubulin were silenced with control oligos or with KIF3A and KIF3B oligos and imaged every 5 min for 15 h. This allowed both a morphological analysis of mitotic cells, by looking at spindle assembly, chromosome alignment and segregation of sister chromatids in the daughter cells, and a quantification of the time that the dividing cells spend in the various step of mitosis, from prophase to anaphase. We found that functional ablation of Kinesin-2 severely delayed the duration of prophase (Figure 4A,B and Supplementary movies M5 and M6) strikingly recalling the defect caused by RAB5 depletion [21,25]. Intriguingly, we observed that RAB5 endosomes are associated with the invaginations of the nuclear membrane during progression of prophase into prometaphase (Supplementary Figure S3) suggesting that, as previously proposed [25], they might assist the release of nuclear membrane components into the endosomal/ER system.

The extended duration of prophase in the Kinesin-2 KD cells was accompanied by defects in the disassembly of the nuclear lamina (Figure 4C,D). Indeed, by comparing cells in prophase with similar degree of chromosome condensation, we noticed that the lamina was thicker and mostly not disassembled in the Kinesin-2 KD cells (Figure 4C,D), phenocopying the defect observed in absence of RAB5 [21,25] (Figure 4C,D).

Measuring the duration of the other mitotic steps in the Kinesin-2 KD cells, we found that prometaphase was marginally prolonged while metaphase was slightly shorter compared to control (Figure 4A), suggesting reduced robustness of the mitotic checkpoint. However, macroscopic alterations in spindle assembly were not found in the majority of Kinesin-2 depleted cells (Supplementary Figures S2A and S4A and movie M4). The most representative defect observed upon ablation of Kinesin-2 was an enlargement of the metaphase plate and only few cells showing severe chromosome uncongression or tripolar spindles were detected in our cell model system (Supplementary Figure S4A,B). Altogether these findings point to the involvement of Kinesin-2 in nuclear envelope breakdown at the onset of mitosis.

**Figure 5:**
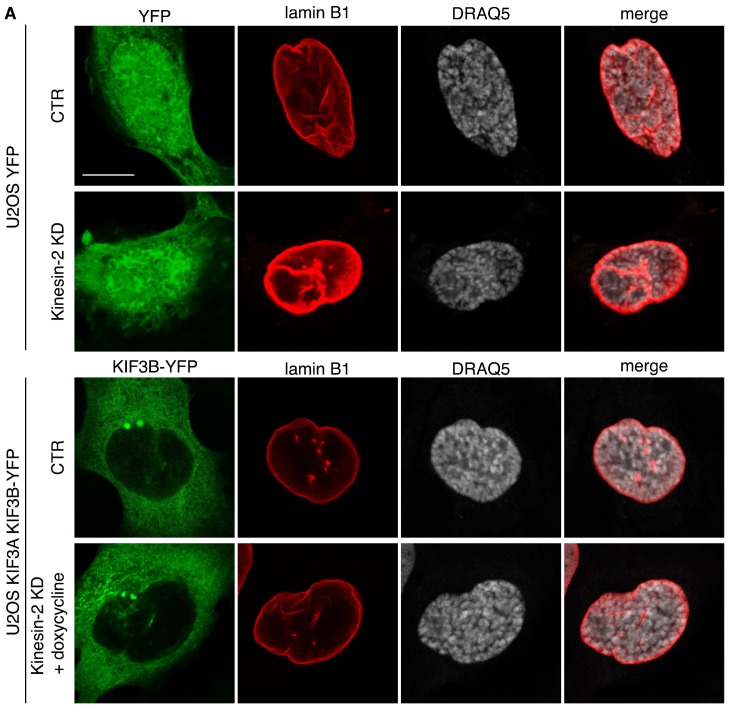

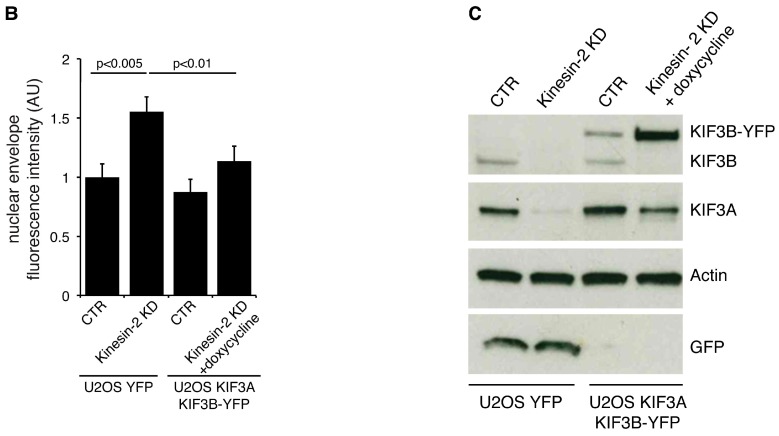
Defective lamina disassembly in Kinesin-2 KD cells is rescued by expression of silencing-resistant KIF3A and KIF3B. U2OS cells stably expressing the YFP empty vector (U2OS YFP) or the silencing-resistant KIF3A and KIF3B-YFP mutants (U2OS KIF3A KIF3B-YFP), indicated on the left, were silenced with control oligo (CTR) or with KIF3A and KIF3B oligos (Kinesin-2 KD). Expression of the silencing-resistant KIF3A mutant in the KIF3B-YFP cells was achieved by doxycycline treatment for 72 h. (**A**) Representative confocal images show epifluorescence of YFP or YFP-KIF3B in green, anti-lamin B1 antibody staining in red, and nuclei stained with DRAQ5 (in gray). (**B**) Quantification of lamin B1 staining in the samples treated as in (**A**) bar graph represents mean values ± SEM normalized over control, from three independent experiments, *n* = 15 cells/condition. (**C**) Total cellular lysates from cells treated as in A were immunoblotted as shown on the right (the band corresponding to KIF3B-YFP is also indicated).

To validate the specificity of the effects scored upon Kinesin-2 silencing we performed rescue experiments. To this end we generated stable U2OS cells expressing the silencing resistant version of both KIF3A (whose expression was driven by a doxycycline-inducible construct) and KIF3B (tagged with YFP, KIF3B-YFP). We silenced endogenous KIF3A and KIF3B in these cells as well as in U2OS control cells expressing the YFP empty vector and we evaluated lamina disassembly at prophase. While silencing of both motors increased the thickness of the lamina in control YFP-expressing cells at prophase, re-expression of the silencing resistant KIF3A and KIF3B mutants rescued normal lamina width confirming the specificity of the knockdown phenotype (Figure 5A,B). Efficient silencing of endogenous Kinesin-2 and expression of silencing-resistant KIF3A (driven by doxycycline) and of KIF3B-YFP in control and silenced cells was confirmed by Western blotting (Figure 5C).

## 3. Discussion

Early endosomes are highly dynamic vesicles; they move bidirectionally at variable speed. The complex array of motility properties displayed by these vesicles likely reflects the involvement of several distinct motors that globally contribute to the homeostasis of this heterogeneous compartment. Endosome positioning and transport within the cell have relevant functional implications. Even if early endosomes are dispersed through the cytoplasm, their transport-dependent functions do not occur at random. This is the case regarding the polarized transport of endosomes during directed cell migration [26] and of maturation of early endosomes into late endosomes, a process that is inhibited by KIF16B-mediated transport [8]. However, few transporters have been described so far to control early endosomes movement and positioning [5].

Here we propose Kinesin-2 as one of the motors that participates to the motility of the early endosomal compartment. Our findings point to the involvement of this motor complex preferentially in the long-range movement of early endosomes since very few vesicles have been found to travel 20 μm far from the original position in absence of Kinesin-2. The abrupt drop in the percentage of endosomes that display this type of movement suggests that Kinesin-2 is responsible for moving a specific pool of endosomes.

KIF3A has been found to associate with RAB proteins [27,28]. In adipocytes stimulated with insulin, KIF3A interacts with RAB4 and drives recycling of the glucose transporter GLUT4 to the cell surface [28]. In these cells, KIF3A does not interact with RAB5 and is not required for GLUT4 endocytosis [28]. However, RAB4 and RAB5 are concomitantly present on endosomes in distinct membrane microdomains that are connected by common RAB4/RAB5 effectors [29,30,31]. These divalent RAB effectors control RAB activation and coordinate protein sorting and recycling [29,31]. It is therefore tempting to speculate that Kinesin-2 moves the RAB5 endosome for maturation or conversion into the recycling pathway, without interacting directly with this GTPase.

At the onset of mitosis, RAB5 endosomes cluster around the spindle poles [21,24]. In human cells, as mitosis proceeds, these clusters disperse and a pool of endosomes can be found within the spindle moving bidirectionally along spindle microtubules [21]. Whether these vesicles have a specific role during mitosis is unknown, however the present study reveals that they are positive for the APPL1 endosomal protein which usually marks a stable cargo sorting compartment [4].

An evolutionary conserved function of RAB5 is to control entry into mitosis by regulating the timing of nuclear envelope breakdown [21,24,25]. The outer nuclear membrane is connected to the continuous network of tubules and sheets that form the endoplasmic reticulum (ER). During nuclear envelope breakdown, some nuclear membrane proteins redistribute into the membrane system of the ER [32]. In *Caenorabditis elegans* embryos, functional ablation of RAB5 results in retention of B-type Lamin at the nuclear membrane and delayed nuclear envelope breakdown [25]. This is observed also in human cells [21,25] and in the “semi-open” mitosis of *Drosophila* cells where dispersal of Lamin is affected in the absence of RAB5 [24]. Here we found that ablation of Kinesin-2 causes a similar phenotype leading us to hypothesize that Kinesin-2 might be involved in the RAB5-dependent pathway that participates to nuclear envelope breakdown and lamina disassembly.

In addition to the defect observed during prophase, RAB5 depletion impairs progression through mitosis causing chromosome uncongression and unbalanced segregation in the daughter cells both in human and *Drosophila* systems [21,24].

These severe mitotic defects were not recapitulated by Kinesin-2 ablation. However it is of note that depletion of the three RAB5 isoforms has profound effects on the entire endo–lysosomal system [1]. In absence of RAB5, biogenesis of early endosomes is defective, and also formation of late endosomes, which originate from conversion of early endosomes, is affected [1]. Differently, in Kinesin-2 KD cells the protein levels of markers of these endosomal compartments are unaltered suggesting that, while Kinesin-2 is responsible for moving and positioning these vesicles, it does not interfere with their biogenesis and this might account for the milder mitotic phenotype.

Overall, our findings point to a role for Kinesin-2 in the long-range movement of RAB5 endosomes during interphase. At the onset of mitosis, Kinesin-2 mediated transport controls timing of nuclear envelope breakdown and localization of RAB5 endosomes at spindle. Several open questions remain to be addressed: Which are the cargoes transported by the spindle-associated RAB5 endosomes, or whether they are connected with the ER/nuclear envelope.

## 4. Material and Methods

### 4.1. Cells Culture and Expression Vectors

U2OS cells, obtained from ATCC, were grown in DMEM (Sigma, Darmstadt, Germany) supplemented with 1% glutamine (Sigma) and 10% FBS (Euroclone, Pero (MI), Italy) or, for cells stably expressing the inducible construct of KIF3A, 10% FBS TET ON (Clontech, Mountain View, CA, USA). To induce KIF3A expression cells were treated with doxycycline 2.5 μg/mL for 72 h. Cells were periodically tested and resulted negative for mycoplasma contamination with VenorGM kit (Minerva BioLabs, Berlin, Germany).

U2OS stable cell populations expressing YFP-RAB5 (the RAB5A isoform) and RFP-α-tubulin or H2B-GFP and RFP-α-tubulin were described in [21].

In RNAi experiments, siRNA oligos from Dharmacon (KIF3A: UAUCGUAACUCUAAACUGA; KIF3B: GGAUAUAAGAGACCAUUGA; RAB5A: AGGAAUCAGUGUUGUAGUAUU; RAB5B: GAAAGUCAAGCCUGGUAUUUU; RAB5C: CAAUGAACGUGAACGAAAUUU; CTR: ON-TARGET plus Nontargeting siRNA #1 (Catalog item D-001810-01-20)) were transfected using RNAiMAX (Invitrogen, Carlsbad, CA, USA) following the manufacturer instructions for reverse transfection. Equal number of cells was plated in presence of siRNA oligos (CTR or KIF3A), and a second round of transfection was repeated the day after on the adherent cells. The KIF3B siRNA oligo was added only during the second round of transfection. Cells were harvested 48 h later. In case of RAB5 silencing, cells were transfected twice with oligos specific for the 3 RAB5 isoforms and harvested 48 h later.

The inducible KIF3A construct was generated by recombinant PCR using the GFP-KIF3A construct kindly provided by Dr. Akiyama [22] mutating 3 nucleotides in the sequence targeted by the silencing oligo without affecting the amino acid sequence, and cloned in the tetracycline-inducible (TET ON) vector pSG213. U2OS cells were transfected with this construct and stable population was selected by puromycin treatment. Next, this cell population was transfected with the silencing-resistant construct of KIF3B-YFP that was generated by gene synthesis (Eurofins, Brussels, Belgium) and cloned in pEYFP-N1. Constructs were sequence verified and details are available upon request. Stable transfectants were selected with neomycin.

### 4.2. Antibodies

Antibodies used were purchased from Santa Cruz (Heidelberg, Germany): EEA1, lamin B1; Abcam (Cambridge, MA, USA): KIF3B, actin, α-tubulin, anti-GFP; Sigma: vinculin, α-tubulin, Lamp1; BD (Franklin Lakes, NJ, USA): KAP3A, RAB5, KIF3A; Antibodies Incorporated (Davis, CA, USA): anti human centromere antibody (ACA). The APPL1 antibody was a kind gift from Marino Zerial’s laboratory (Max Planck Institute of Molecular Cell Biology and Genetics, Dresden, Germany) [33].

### 4.3. Immunofluorescence

For APPL1 and EEA1 stainings, U2OS cells stably expressing YFP-RAB5 were fixed with PAF 4% for 10 min at room temperature, washed 3 times with PBS and permeabilized for 30 min in PBS saponine 0.1%, BSA 2%. Both primary and secondary antibodies were diluted in PBS saponine 0.1%, BSA 0.2%.

For both α-tubulin and lamin B1 stainings, U2OS cells were fixed in PAF 4% for 10 min, permeabilized with PBS 0.1% Triton X-100 for 10 min and stained as previously described [34]. For KIF3A stainings, U2OS cells were fixed with cold methanol for 2 min on ice, washed 3 times with PBS, and saturated in PBS BSA 3% for 30 min at room temperature. Both primary and secondary antibodies were diluted in PBS BSA 1%.

For nuclear staining with DRAQ5 (Invitrogen), the die was added with the secondary antibodies following the manufacturer’s instructions.

Confocal analysis was performed with the confocal Leica SP8 AOBS microscope using a 63× immersion objective. Images in Figure 4C and Figure S4B were acquired using a Leica TCS SP5 AOBS microscope with a 63× immersion objective.

Colocalization between RAB5 and either EEA1 or APPL1 was analyzed with ImageJ by creating a mask around the spindle and measuring the overlap between the two channels both inside and outside the region identified by the mask using the JacoP plugin.

The number of RAB5 vesicles located on the spindle (presented in Figure S2) was measured with ImageJ. A mask was designed around the spindle and the number of RAB5 positive vesicles was automatically calculated using the analyze particles tools.

Lamina thickness was measured using ImageJ. Three lines were drawn perpendicular to the nuclear envelope and the intensity of the signal was evaluated using the measure tool of ImageJ as described in [21].

### 4.4. Live Imaging and Analysis

Analysis of RAB5 endosome motility in interphase was performed on a Leica SP8 AOBS microscope with a 100× (N.A. = 1.4) immersion objective keeping U2OS cells stably expressing YFP-RAB5 at 37 °C, 5% CO_2_ for the entire duration of the experiment. Cells were imaged for ~2 min at a speed of 3.83 frames per second. The motility properties of each vesicle were analyzed by evaluating its maximal displacement from its initial position during the observation time T = 2 min, which was defined as:

$$d_{\max}=\underset{t\in \left[0,\;T\right]}{\max}\left|x_{cm}\left(t\right)-x_{cm}\left(0\right)\right|$$

where $x_{cm}$ is the coordinate vector of its center of mass and the operator | | indicates the Euclidean norm. Vesicle positions were obtained by analyzing the experimental movies with computational routines properly encoded in Matlab (The MathWorks, Natick, MA, USA). The use of this parameter allowed us to avoid the bias due to possible back and forth motions, as stated in several experimental and computational works [35,36].

The wind-rose plots were generated by connecting, with a smooth cubic curve, the positions of the center of mass of randomly chosen vesicles taken at 0.26 s-time intervals until T = 2 min overlying the starting coordinates at the origin of the graph for a representative cell either in the control or in the silenced for Kinesin-2 condition.

Furthermore the movies were analyzed using the MetaMorph tracker plugin “track objects” to evaluate the mean velocity of the vesicles in control cells versus cells silenced for Kinesin-2.

For the analysis of RAB5 endosomes on the mitotic spindle, time lapse of cells stably expressing YFP-RAB5 and RFP–α-tubulin was done with an UltraVIEW VoX spinning disk confocal unit (PerkinElmer, Waltham, MA, USA) equipped with an inverted Nikon Eclipse Ti microscope and a Yokogawa CSU-X1 scanning head driven by Volocity software (Improvision; PerkinElmer). A 60× oil immersion objective (N.A. = 1.42) was used.

The movies are the result of a Z-projection of 7 slices (corresponding to 3 μm) taken in the equatorial region of the spindle. For each cell acquisition was performed every 3 s for ∼4 min. During the experiment cells were kept at 37 °C at a control atmosphere of 5% CO_2_. Movies were then analyzed using a homemade macro in ImageJ. Briefly the macro created a region of interest around the spindle calculating the number of vesicles on the spindle, relative to the total number of vesicles, at each time point.

For the evaluation of the mitotic delay, living U2OS cells stably expressing H2B-GFP and RFP–α-tubulin were acquired using a 40× objective on a Leica AF 6000LX workstation: 10 fields per condition were acquired every 5 min for 15 h performing a 8 μm *z*-stack. During the experiment, cells were kept at 37 °C at a control atmosphere of 5% CO_2_. After acquisition movies were analyzed evaluating the time that each mitotic cell spent in the various steps from prophase to anaphase.

### 4.5. Statistical Analysis

Statistical analyses were done with GraphPad (La Jolla, CA, USA) using the Wilcoxon test unless otherwise mentioned.

## Supplementary Materials

Supplementary materials can be found at http://www.mdpi.com/1422-0067/19/9/2575/s1.
