# Supplementary material for: Kinesin-2 Controls the Motility of RAB5 Endosomes and Their Association with the Spindle in Mitosis
Source: Int J Mol Sci. 2018 Aug 30;19(9):2575. doi: 10.3390/ijms19092575 (PMC6163544; doi:10.3390/ijms19092575)
Supplement: Supplementary file 1 [file ijms-19-02575-s001.zip › Supplementary Materials.pdf]

SUPPLEMENTARY DATA

Legends to Supplementary Figures, Table and Movies

<table>
<tr><td colspan="3">Y2H screen - Human KIAA0019(326-839) vs PLA_RP (Human Placenta Random Primed 4)</td></tr>
<tr><td></td><td></td><td></td></tr>
<tr><td>Clone Name</td><td>Contig(s) Name</td><td>Gene Name (Best Match)</td></tr>
<tr><td>hgx1165v1_pB27A-41</td><td>10366186</td><td>hACTN4; [gi|34452697|ref|NM_004924.3| Homo sapiens actinin, alpha 4 (ACTN4), mRNA]</td></tr>
<tr><td>hgx1165v1_pB27A-24</td><td>10366140</td><td>hEIF3S10; htranslation initiation factor 3 largesubunit; [gi|4503508|ref|NM_003750.1| Homo sapiens eukaryotic translation initiation factor 3, subunit 10 theta, 150/170kDa (EIF3S10), mRNA]</td></tr>
<tr><td>hgx1165v1_pB27A-48</td><td>10366189</td><td>hEPS8R1; [gi|21264607|ref|NM_133180.1| Homo sapiens epidermal growth factor receptor pathway substrate 8-related protein 1 (EPS8R1), transcript variant 1, mRNA]</td></tr>
<tr><td>hgx1165v1_pB27A-1</td><td>10366193</td><td>hFBXL11; [gi|16306579|ref|NM_012308.1| Homo sapiens f-box and leucine-rich repeat protein 11 (FBXL11), mRNA]</td></tr>
<tr><td>hgx1165v1_pB27A-4</td><td>10366202</td><td>hHEY1; hHES-related repressor protein 2HERP2; [gi|20149602|ref|NM_012258.2| Homo sapiens hairy/enhancer-of-split related with YRPW motif 1 (HEY1), mRNA]</td></tr>
<tr><td>hgx1165v1_pB27A-39</td><td>10366202</td><td>hHEY1; hHES-related repressor protein 2HERP2; [gi|20149602|ref|NM_012258.2| Homo sapiens hairy/enhancer-of-split related with YRPW motif 1 (HEY1), mRNA]</td></tr>
<tr><td>hgx1165v1_pB27A-17</td><td>10366147</td><td>hHSPC063; [gi|56711303|ref|NM_014155.2| Homo sapiens HSPC063 protein (HSPC063), mRNA]</td></tr>
<tr><td>hgx1165v1_pB27A-10</td><td>10366147</td><td>hHSPC063; [gi|56711303|ref|NM_014155.2| Homo sapiens HSPC063 protein (HSPC063), mRNA]</td></tr>
<tr><td>hgx1165v1_pB27A-33</td><td>10366147</td><td>hHSPC063; [gi|56711303|ref|NM_014155.2| Homo sapiens HSPC063 protein (HSPC063), mRNA]</td></tr>
<tr><td>hgx1165v1_pB27A-5</td><td>10366147</td><td>hHSPC063; [gi|56711303|ref|NM_014155.2| Homo sapiens HSPC063 protein (HSPC063), mRNA]</td></tr>
<tr><td>hgx1165v1_pB27A-12</td><td>10366147</td><td>hHSPC063; [gi|56711303|ref|NM_014155.2| Homo sapiens HSPC063 protein (HSPC063), mRNA]</td></tr>
<tr><td>hgx1165v1_pB27A-6</td><td>10366191</td><td>hKIBRA; [gi|29789057|ref|NM_015238.1| Homo sapiens KIBRA protein (KIBRA), mRNA]</td></tr>
<tr><td>hgx1165v1_pB27A-13</td><td>10366184</td><td>hKIF3A; [gi|46852173|ref|NM_007054.4| Homo sapiens kinesin family member 3A (KIF3A), mRNA]</td></tr>
<tr><td>hgx1165v1_pB27A-47</td><td>10366207 / 10366144</td><td>hMAGI-3; [gi|29568112|ref|NM_020965.2| Homo sapiens membrane-associated guanylate kinase-related (MAGI-3) (MAGI-3), mRNA]</td></tr>
<tr><td>hgx1165v1_pB27A-3</td><td>10366207 / 10366144</td><td>hMAGI-3; [gi|29568112|ref|NM_020965.2| Homo sapiens membrane-associated guanylate kinase-related (MAGI-3) (MAGI-3), mRNA]</td></tr>
<tr><td>hgx1165v1_pB27A-11</td><td>10366133</td><td>hMBD1; [gi|21464116|ref|NM_015846.2| Homo sapiens methyl-CpG binding domain protein 1 (MBD1), transcript variant 1, mRNA]</td></tr>
<tr><td>hgx1165v1_pB27A-23</td><td>10366182</td><td>hMSC; [gi|6996017|ref|NM_005098.2| Homo sapiens musculin (activated B-cell factor-1) (MSC), mRNA]</td></tr>
<tr><td>hgx1165v1_pB27A-37</td><td>10366212</td><td>hPHGDHL1; [gi|31342615|ref|NM_177967.2| Homo sapiens phosphoglycerate dehydrogenase like 1 (PHGDHL1), mRNA]</td></tr>
<tr><td>hgx1165v1_pB27A-22</td><td>10366135 / 10366137</td><td>hPIAS2; [gi|56699459|ref|NM_173206.2| Homo sapiens protein inhibitor of activated STAT, 2 (PIAS2), transcript variant alpha, mRNA]</td></tr>
<tr><td>hgx1165v1_pB27A-21</td><td>10366138 / 10366195</td><td>hPIASY; hUnknown (protein forMGC:35296); [gi|24850132|ref|NM_015897.2| Homo sapiens protein inhibitor of activated STAT protein PIASy (PIASY), mRNA]</td></tr>
<tr><td>hgx1165v1_pB27A-16</td><td>10366195</td><td>hPIASY; hUnknown (protein forMGC:35296); [gi|24850132|ref|NM_015897.2| Homo sapiens protein inhibitor of activated STAT protein PIASy (PIASY), mRNA]</td></tr>
<tr><td>hgx1165v1_pB27A-18</td><td>10366195</td><td>hPIASY; hUnknown (protein forMGC:35296); [gi|24850132|ref|NM_015897.2| Homo sapiens protein inhibitor of activated STAT protein PIASy (PIASY), mRNA]</td></tr>
<tr><td>hgx1165v1_pB27A-40</td><td>10366195</td><td>hPIASY; hUnknown (protein forMGC:35296); [gi|24850132|ref|NM_015897.2| Homo sapiens protein inhibitor of activated STAT protein PIASy (PIASY), mRNA]</td></tr>
<tr><td>hgx1165v1_pB27A-42</td><td>10366142 / 10366188</td><td>hSF3B1; [gi|54112116|ref|NM_012433.2| Homo sapiens splicing factor 3b, subunit 1, 155kDa (SF3B1), transcript variant 1, mRNA]</td></tr>
<tr><td>hgx1165v1_pB27A-15</td><td>10366205</td><td>hTEAD3; [gi|42490752|ref|NM_003214.2| Homo sapiens TEA domain family member 3 (TEAD3), mRNA]</td></tr>
<tr><td>hgx1165v1_pB27A-14</td><td>10366158</td><td>hTTF1; [gi|33356178|ref|NM_007344.2| Homo sapiens transcription termination factor, RNA polymerase I (TTF1), mRNA]</td></tr>
<tr><td>hgx1165v1_pB27A-27</td><td>10366210</td><td>hUTY; [gi|33188428|ref|NM_182660.1| Homo sapiens ubiquitously transcribed tetratricopeptide repeat gene, Y chromosome (UTY), transcript variant 1, mRNA]</td></tr>
<tr><td>hgx1165v1_pB27A-34</td><td>10366174</td><td>hUnknown (protein forMGC:26123); [gi|21040474|gb|BC030580.1| Homo sapiens, clone MGC:26123 IMAGE:4823171, mRNA, complete cds]</td></tr>
<tr><td>hgx1165v1_pB27A-20</td><td>10366174</td><td>hUnknown (protein forMGC:26123); [gi|21040474|gb|BC030580.1| Homo sapiens, clone MGC:26123 IMAGE:4823171, mRNA, complete cds]</td></tr>
<tr><td>hgx1165v1_pB27A-2</td><td>10366174</td><td>hUnknown (protein forMGC:26123); [gi|21040474|gb|BC030580.1| Homo sapiens, clone MGC:26123 IMAGE:4823171, mRNA, complete cds]</td></tr>
<tr><td>hgx1165v1_pB27A-30</td><td>10366174</td><td>hUnknown (protein forMGC:26123); [gi|21040474|gb|BC030580.1| Homo sapiens, clone MGC:26123 IMAGE:4823171, mRNA, complete cds]</td></tr>
<tr><td>hgx1165v1_pB27A-44</td><td>10366174</td><td>hUnknown (protein forMGC:26123); [gi|21040474|gb|BC030580.1| Homo sapiens, clone MGC:26123 IMAGE:4823171, mRNA, complete cds]</td></tr>
<tr><td>hgx1165v1_pB27A-38</td><td>10366174</td><td>hUnknown (protein forMGC:26123); [gi|21040474|gb|BC030580.1| Homo sapiens, clone MGC:26123 IMAGE:4823171, mRNA, complete cds]</td></tr>
<tr><td>hgx1165v1_pB27A-36</td><td>10366174</td><td>hUnknown (protein forMGC:26123); [gi|21040474|gb|BC030580.1| Homo sapiens, clone MGC:26123 IMAGE:4823171, mRNA, complete cds]</td></tr>
<tr><td>hgx1165v1_pB27A-28</td><td>10366153</td><td>hZNF292; [gi|51465426|ref|XM_048070.4| PREDICTED: Homo sapiens zinc finger protein 292 (ZNF292), mRNA]</td></tr>
<tr><td>hgx1165v1_pB27A-29</td><td>10366162 / 10366153</td><td>hZNF292; [gi|51465426|ref|XM_048070.4| PREDICTED: Homo sapiens zinc finger protein 292 (ZNF292), mRNA]</td></tr>
<tr><td>hgx1165v1_pB27A-31</td><td>10366162 / 10366153</td><td>hZNF292; [gi|51465426|ref|XM_048070.4| PREDICTED: Homo sapiens zinc finger protein 292 (ZNF292), mRNA]</td></tr>
<tr><td>hgx1165v1_pB27A-43</td><td>10366162 / 10366153</td><td>hZNF292; [gi|51465426|ref|XM_048070.4| PREDICTED: Homo sapiens zinc finger protein 292 (ZNF292), mRNA]</td></tr>
<tr><td>hgx1165v1_pB27A-9</td><td>10366170</td><td>hhairy/enhancer-of-split related with YRPWmotif-like; hHEYL; hhairy-related transcription factor3; [gi|19923414|ref|NM_014571.2| Homo sapiens hairy/enhancer-of-split related with YRPW motif-like (HEYL), mRNA]</td></tr>
<tr><td>hgx1165v1_pB27A-32</td><td>10366199</td><td>hthyroid autoantigen 70kD (Kuantigen); hG22P1; [gi|20070134|ref|NM_001469.2| Homo sapiens thyroid autoantigen 70kD (Ku antigen) (G22P1), mRNA]</td></tr>
<tr><td>hgx1165v1_pB27A-35</td><td>10366199</td><td>hthyroid autoantigen 70kD (Kuantigen); hG22P1; [gi|20070134|ref|NM_001469.2| Homo sapiens thyroid autoantigen 70kD (Ku antigen) (G22P1), mRNA]</td></tr>
<tr><td>hgx1165v1_pB27A-25</td><td>10366208</td><td>unknown; [prey516561 - Human - GenMatch]</td></tr>
<tr><td>hgx1165v1_pB27A-7</td><td>10366131</td><td>unknown; [gi|34193108|gb|BC040990.2| Homo sapiens cDNA clone IMAGE:4817441, partial cds]</td></tr>
</table>

Supplementary Table S1

Supplementary Table S1. USP6NL Yeast Two Hybrid interactions

The C-terminal region of the RAB5 GTPase-activating protein USP6NL (starting from amino acid position 326 to the end of the protein) was used as bait in Yeast Two Hybrid experiments done with a placenta library of preys. Experiments were performed by HYBRIGENICS. Using this method, we confirmed previously published interactions between USP6NL and Eps8 [Epidermal Growth Factor receptor pathway substrate 8-related protein 1 (EPS8R1)] and ACTN4 (actinin, alpha 4). Novel putative interactors were found, including KIF3A.

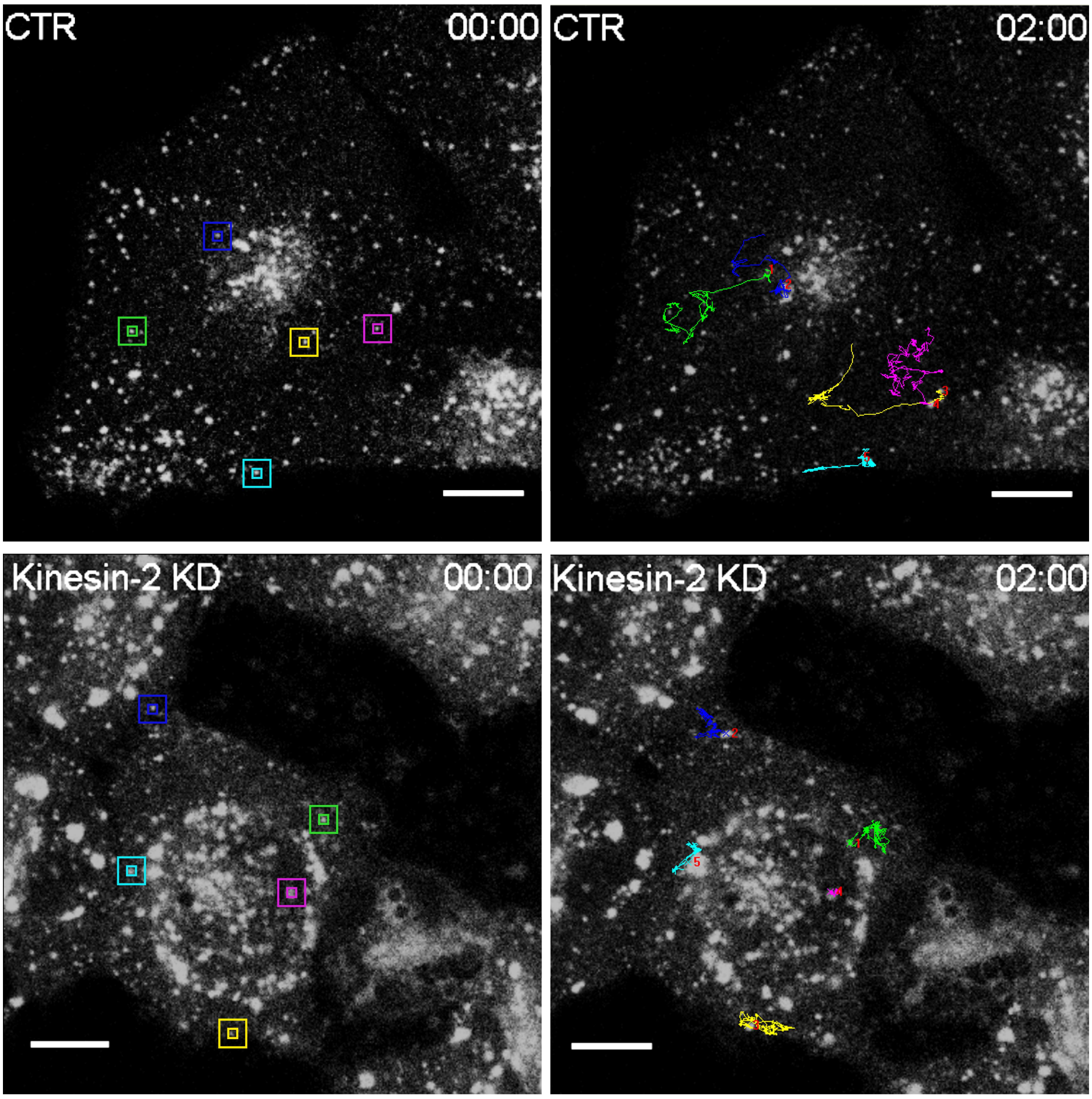

Supplementary Figure S1. Tracks travelled by RAB5 endosomes in control and Kinesin-2 silenced cells. Snapshots taken at time 0 and 2 min from movies M1 (control cell, CTR, top row) and M2 (Kinesin-2 silenced cell, bottom row). Five vesicles have been chosen in each movie at time 0 and they are shown encased by squares (left column). The tracks they travelled, drawn by the program, are shown in the images corresponding to time point 2 min (end of the movies) on the right column. Bar here and in the following pictures is 10 μm.

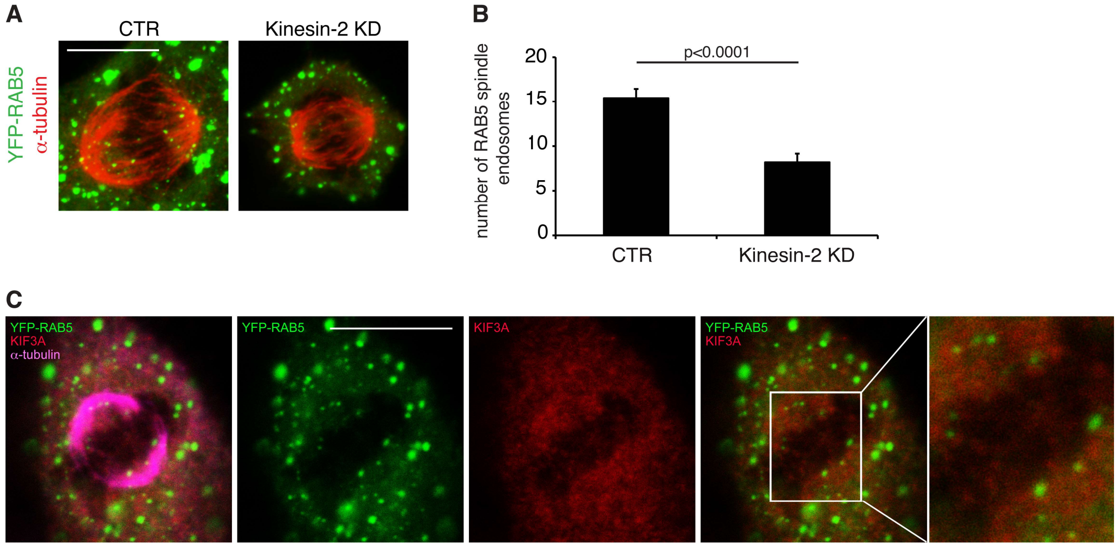

Supplementary Figure S2. Localization of RAB5 endosomes in mitotic cells evaluated upon fixation

A) Maximum projection of confocal images taken in the equatorial region of the cell (6 slices, for a total thickness of 3 μm) of U2OS cells stably expressing YFP-RAB5 silenced with control oligo (CTR) or with KIF3A and KIF3B oligos (Kinesin-2 KD) stained with α-tubulin. B) Bar graph representing the number of RAB5-positive vesicles within the spindle in cells silenced as on bottom. The number of vesicles was obtained using the ImageJ analyze particles tool after creating a Region Of Interest (ROI) around the mitotic spindle. At least 20 cells per condition from 3 independent experiments were analyzed (means ± sem). C) Confocal representative images of a metaphase U2OS cell expressing YFP-RAB5 (in green) stained with anti-KIF3A (in red) and α-tubulin antibodies (in magenta). In the YFP-RAB5 and KIF3A merged image a region corresponding to the spindle is boxed and magnified in the rightmost panel.

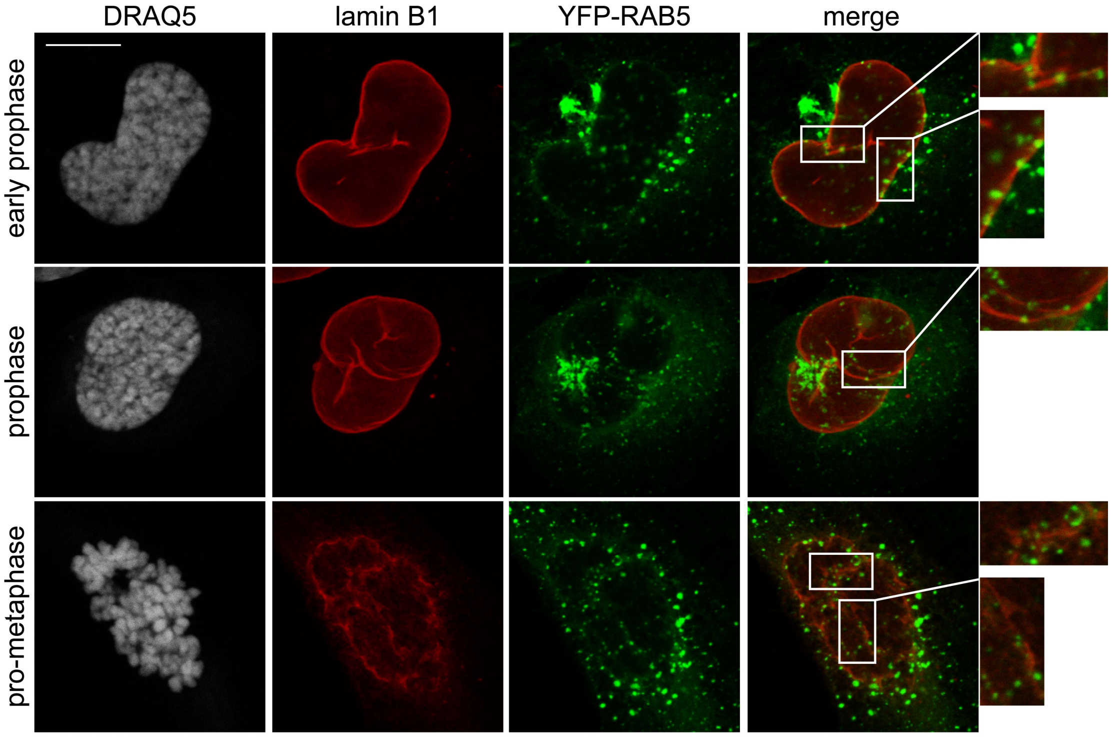

Supplementary Figure S3. Localization of RAB5 endosomes during nuclear envelope breakdown

Confocal sections of U2OS cells expressing YFP-RAB5 (in green) stained with anti-lamin B1 antibody (in red). Nuclei are revealed by DRAQ5 (in gray). Regions displaying invaginations of the nuclear membrane are boxed in merge and magnified in the insets on the right.

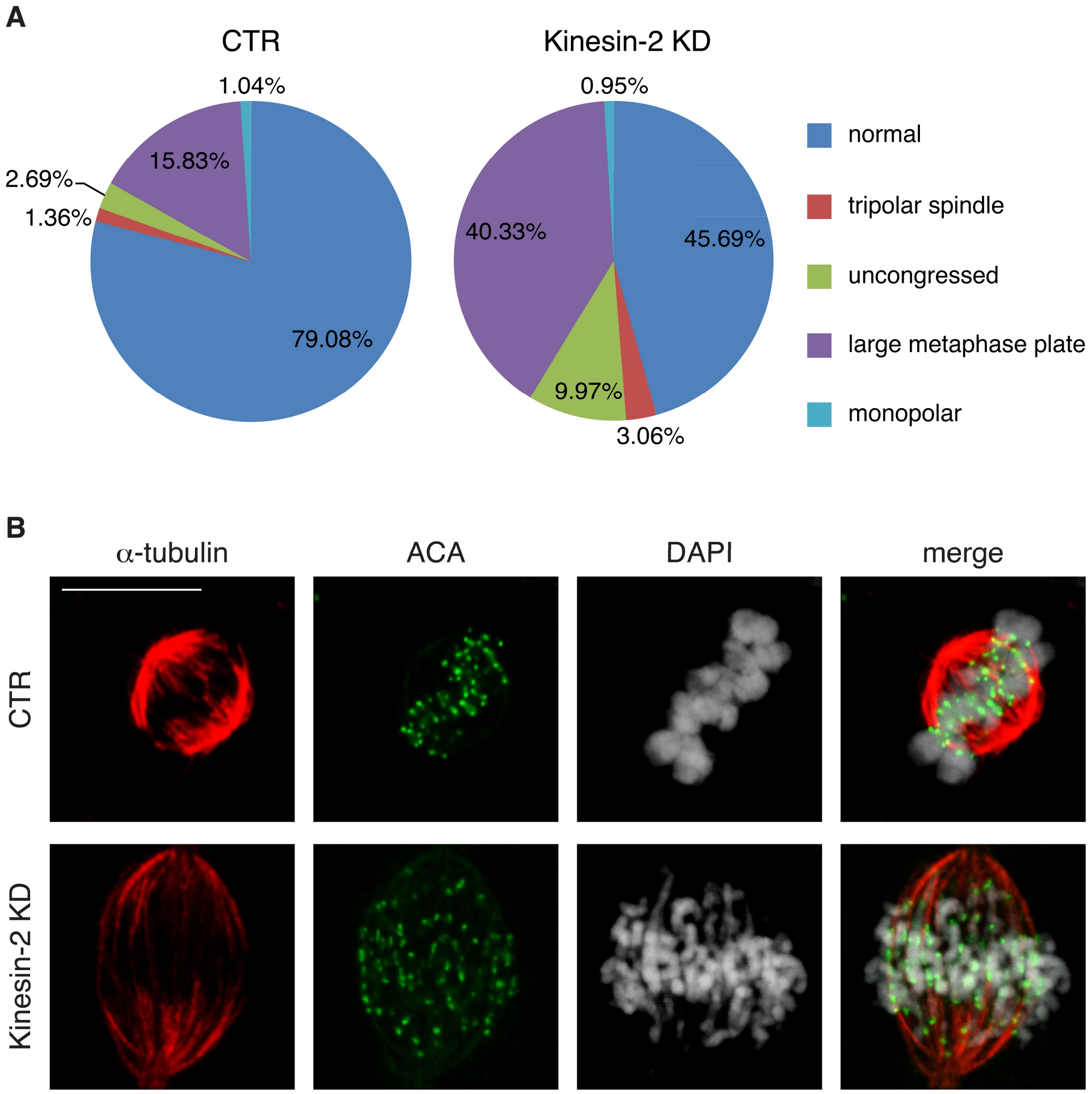

Supplementary Figure S4. Mitotic defects classification in the Kinesin-2 silenced cells

A) Evaluation of mitotic defects (indicated in the legend on the right) in U2OS cells silenced with control oligo (CTR) or with KIF3A and KIF3B oligos (Kinesin-2 KD). Silenced cells were treated with MG132 (Calbiochem) for 2 hours to block mitotic cells at metaphase, fixed and stained with anti-α-tubulin and ACA, a marker of kinetochores, antibodies and DAPI. 283 mitotic cells were considered in the CTR condition, 301 cells in the Kinesin-2 KD condition. B) Maximum Z-stack projection of confocal images of cells treated as in (A)

and stained with α-tubulin (red), ACA (green) and DAPI (gray). Merged images are also shown.

Movies M1 and M2. Kinesin-2 participates to RAB5-endosomes motility

U2OS cells stably expressing YFP-RAB5 silenced with control oligo (CTR, M1) or with KIF3A and KIF3B oligos (Kinesin-2 KD, M2) were acquired with a Leica SP8 AOBS microscope with a 100X immersion objective for 2 minutes. Frames were captured every 0.26 sec. Time is shown in seconds. Bar is 10 μm.

Movies M3 and M4 Kinesin-2 depletion impairs localization of RAB5-endosomes within the spindle

U2OS cells stably expressing YFP-RAB5 and RFP-α-tubulin were silenced with control oligo (CTR, M3) or with KIF3A and KIF3B oligos (Kinesin-2 KD, M4). Time lapse was performed with an UltraVIEW VoX spinning disk confocal unit (PerkinElmer) equipped with an inverted Nikon Eclipse Ti microscope and a Yokogawa CSU-X1 scanning head driven by Volocity software (Improvision; PerkinElmer). The movies are the result of a Z-projection of 7 slices (corresponding to 3 μm) taken in the equatorial region of the spindle. Images were acquired every 3 seconds for ~4 minutes. Bar is 10 μm.

Movies M5 and M6 Kinesin-2 depletion delays the onset of mitosis

U2OS cells stably expressing H2B-GFP were silenced with control oligo (CTR, M5) or with KIF3A and KIF3B oligos (Kinesin-2 KD, M6) and acquired

every 5 minutes for 15 hours with a Leica AF6000LX fluorescent workstation using a 40X objective. Time is in minutes; t = 0 is defined as the time point at which chromosome condensation becomes evident.
